# Celiac trunk occlusion as a severe complication of atrial fibrillation: A case report

**DOI:** 10.1016/j.radcr.2025.11.015

**Published:** 2025-12-05

**Authors:** Ahmed Bengrad, Bezzazi Rawane, Banana Youssef, Laarouchi Abdennasser, Oussama Anane, Abdellah Rezziki, Adnane Benzirar, Omar El Mahi

**Affiliations:** Department of Vascular Surgery, Mohammed VI University Hospital of Oujda, Mohammed First University of Oujda, Oujda, Morocco

**Keywords:** Celiac trunk occlusion, Acute mesenteric ischemia, Atrial fibrillation, Arterial embolism, Case report

## Abstract

Acute occlusion of the celiac trunk is an exceptional cause of mesenteric ischemia. Although atrial fibrillation is one of the main causes of arterial embolism, its presentation as an isolated celiac trunk embolism is extremely rare and associated with a particularly poor prognosis.

We report the case of a 68-year-old woman with no prior medical history, admitted with severe abdominal pain evolving over 3 days. On admission, she presented in shock, and electrocardiography revealed previously unknown atrial fibrillation. Laboratory tests showed leukocytosis, hyperlactatemia, and signs of multiorgan dysfunction. Abdominal CT angiography demonstrated a thromboembolic occlusion of the celiac trunk with absence of perfusion of the main branches. Emergency thrombectomy was indicated, but the outcome was rapidly fatal with death occurring before surgery.

This case highlights the severity of this rare localization of arterial embolism and underlines the importance of early diagnosis and prompt management.

## Introduction

Acute occlusion of the celiac trunk is an extremely rare event. While acute mesenteric ischemia is most often related to embolism of the superior mesenteric artery, isolated involvement of the celiac trunk is exceptional.

Atrial fibrillation is the most common cause of arterial embolism of cardiac origin. However, its localization to the celiac trunk is unusual and leads to multivisceral ischemia involving the liver, spleen, stomach, and pancreas.

Diagnosis relies on CT angiography, which allows visualization of the occlusion, assessment of its visceral consequences, and guidance for therapeutic management. Nevertheless, diagnostic delay remains the main prognostic factor, with mortality remaining very high in such cases.

We report the case of a patient admitted in shock, revealing an acute celiac trunk occlusion due to previously unknown atrial fibrillation, illustrating the severity of this rare localization and the importance of early diagnosis.

## Case presentation

A 68-year-old woman, with no known medical history, was admitted for severe abdominal pain evolving over 3 days. The pain had appeared abruptly, was diffuse, and progressively worsened, associated with nausea and anorexia.

On admission, the patient was in shock, with a blood pressure of 80/50 mmHg, heart rate of 140 beats/min, respiratory rate of 28 cycles/min, and oxygen saturation of 90% on room air. She was pale and clinically unstable.

Abdominal examination revealed a distended, very painful abdomen with diffuse guarding but no peritoneal rigidity. Bowel sounds were absent.

Electrocardiography showed new-onset rapid atrial fibrillation.

Laboratory investigations revealed:

• Leukocytosis at 18,500/µL,
• C-reactive protein 185 mg/L,
• Metabolic acidosis with lactate at 6.2 mmol/L,
• Signs of multiorgan dysfunction including hepatic cytolysis and early renal failure.

**Fig. 1:**
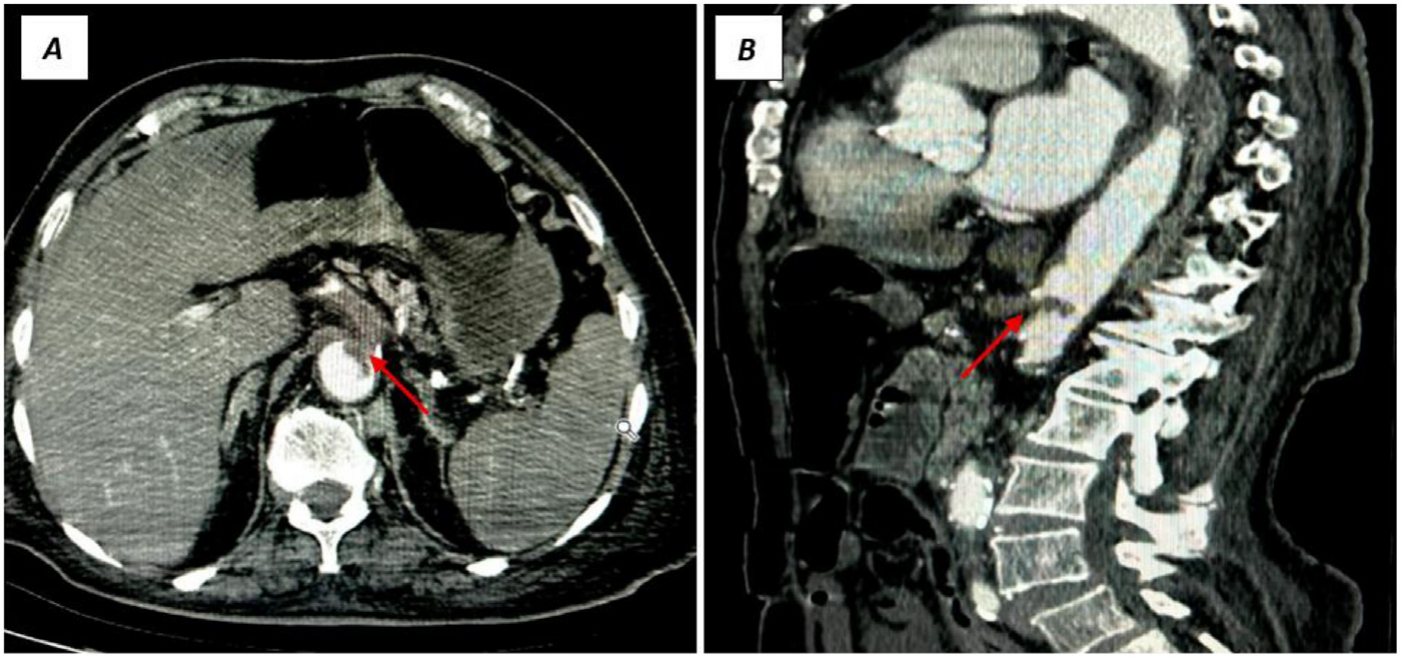
Abdominal CT angiography showing acute occlusion of the celiac trunk. (A) Axial view, (B) Sagittal reconstruction, demonstrating absence of opacification of the celiac trunk (red arrows).

After initial hemodynamic stabilization, an abdominal CT angiography was performed, which demonstrated a thromboembolic occlusion of the celiac trunk with absence of perfusion of the main branches : hepatic, splenic, and left gastric arteries (Fig. 1) .

The diagnosis of acute mesenteric ischemia due to celiac trunk embolism secondary to atrial fibrillation was established. After multidisciplinary consultation, urgent surgical thrombectomy was indicated. Unfortunately, the patient’s outcome was rapidly unfavorable, with death occurring before the intervention could be performed (Fig. 1).

## Discussion

Acute celiac trunk occlusion is a rare entity. While acute mesenteric ischemia most frequently involves the superior mesenteric artery, isolated occlusion of the celiac trunk remains exceptional. It results in multivisceral ischemia because the celiac trunk supplies essential organs such as the liver, spleen, stomach, and pancreas [1,2]. Only a few cases have been reported in the literature, and the mortality rate is particularly high.

Currently, CT angiography is considered the reference examination [3,4]. It allows precise identification of the occlusion site and extent, assessment of visceral perfusion and early ischemic signs, detection of complications such as bowel necrosis or pneumatosis, and guidance for therapeutic management, whether surgical or endovascular [5].

The most important prognostic factor remains the delay in diagnosis. Acute mesenteric ischemia rapidly progresses to necrosis and septic shock. In embolic forms, revascularization should ideally be performed within the first 6-12 hours; beyond 24 hours, mortality can reach 80%-100% [6,7]. Our case illustrates this reality: a 3-day delay before consultation resulted in extensive multivisceral ischemia and irreversible shock, rendering any therapeutic attempt ineffective.

The prognosis of celiac trunk occlusion is therefore particularly poor. Reported mortality exceeds 70% due to the extent of multivisceral ischemia, even with specialized management [2]. Only early diagnosis and rapid revascularization are associated with improved survival [8].

In summary, acute celiac trunk occlusion is a rare but dramatic condition. Diagnosis relies on modern imaging, but prognosis depends primarily on early recognition and prompt management. Our case illustrates the fatal outcome related to major diagnostic delay.

## Conclusion

Acute celiac trunk occlusion is an exceptional localization of mesenteric ischemia, most often embolic in origin secondary to atrial fibrillation. This case underlines the severity of this condition, whose prognosis remains dramatic in the event of delayed diagnosis, emphasizing the importance of clinical suspicion, early imaging, and urgent management to improve survival.

## Author contributions

The authors declare that this is their original work, and all approve the content of this manuscript. They confirm that this manuscript has not been published previously, in any language, in whole or in part, and is not currently under consideration elsewhere.

## Ethical approval

This report did not involve any research protocol and therefore did not require ethical approval.

## Patient consent

Written informed consent for publication of this case report and accompanying images was obtained from the patient’s next of kin.
